# Age-specific early-life gut microbiome associations with eczema and food allergies during early immune development

**DOI:** 10.3389/frmbi.2026.1804117

**Published:** 2026-05-29

**Authors:** Harold Nunez, Timothy J. Straub, Nabeel Imam, David Goad, Noel T. Mueller, Ruben A. T. Mars, Cheryl Sew Hoy, Trillitye Paullin, Kimberley V. Sukhum

**Affiliations:** 1 Seeding Inc., doing business as Tiny Health, Austin, TX, United States; 2 Department of Pediatrics, Section of Nutrition, University of Colorado Anschutz Medical Campus, Aurora, CO, United States; 3 Department of Epidemiology, Colorado School of Public Health, Denver, CO, United States; 4 Division of Gastroenterology and Hepatology, Mayo Clinic, Rochester, MN, United States; 5 Free to Feed, Durham, NC, United States

**Keywords:** atopic march, eczema, food allergies, gut microbiome, short chain fatty acids

## Abstract

**Introduction:**

Eczema and food allergy commonly emerge during infancy and are linked to changes in the gut microbiome, yet it remains unclear when microbiome differences associated with allergic disease first appear during development.

**Methods:**

We analyzed age-stratified shotgun metagenomic data from 97 children aged 4–36 months, including physician-confirmed cases of eczema or food allergy and non-allergic controls, excluding recent antibiotic or probiotic exposure. Microbial taxa, functional pathways, and composite microbiome metrics were evaluated across three developmental stages: early infancy (4–6 months), mid-infancy (6–12 months), and toddlerhood (12–36 months).

**Results:**

Differences between allergic and non-allergic children were minimal before 6 months of age but became more apparent during mid-infancy and persisted into toddlerhood. Allergic conditions were associated with reduced abundance of fiber-fermenting and butyrate-producing taxa, enrichment of facultative and inflammation-associated microbes, lower microbiome maturation scores, and shifts in metabolic and inflammatory functional capacity.

**Discussion:**

These findings suggest that gut microbiome divergence associated with allergic disease becomes more apparent during mid-infancy, highlighting a developmentally relevant period for understanding early immune disruption. The results support further longitudinal and interventional studies aimed at clarifying whether earlier microbiome-targeted strategies may help modify progression along the atopic march.

## Introduction

In the United States, approximately one in ten children has eczema and one in twenty has a diagnosed food allergy, conditions that substantially affect the quality of life for affected families (Lv et al., 2024; Sánchez-Borges et al., 2018; Zablotsky et al., 2023). These allergic conditions are part of a broader developmental sequence known as the atopic march, which often starts with atopic dermatitis (AD; eczema) in infancy, typically around 4–9 months of age, and progresses to food allergy (FA), allergic rhinitis, and asthma (Hill and Spergel, 2018; Hoskinson et al., 2023; Falcon and Caoili, 2023; Wan et al., 2019).

The gut microbiome is a critical regulator of immune development and homeostasis. In early life, it supports the transition from neonatal Th2 dominance toward a more balanced Th1/Th2 immune profile (Henrick et al., 2021). This early life period is marked by rapid microbial and immunological maturation influenced by factors such as cesarean delivery, antibiotic use, and diet (Shao et al., 2019; Stokholm et al., 2018; Sandall et al., 2018; Nunez et al., 2025). In addition, maternal gut microbes and their metabolites during pregnancy may influence infant immune development through metabolite-mediated signaling and early microbial transmission, shaping initial colonization during birth and breastfeeding and potentially contributing to allergy risk (Kalbermatter et al., 2021; Du et al., 2025). Prior studies have identified microbiome-allergy associations (Sbihi et al., 2019; De Filippis et al., 2021), but these studies are limited by lack of metagenomics data which can provide insight into species, strains, and microbial functions related to immune development. Moreover, most studies depend on self-reporting of allergic disease and generally do not stratify by age, despite the rapid developmental changes in microbial ecology that occur across infancy, mid-infancy, and the toddler stages due to major transitions in infant feeding patterns. Because of these limitations, it remains unclear at what developmental stage microbiome differences associated with allergic disease first emerge, and whether these differences align with known windows of immune maturation. This question is important, as it informs the design of targeted strategies to modulate the gut microbiome.

To address this question, we profiled the gut microbiome of children aged four to 36 months, spanning infancy, mid-infancy, and toddler stages using shotgun metagenomic sequencing, focusing on three developmental stages (4–6 months, 6–12 months, and 12–36 months) aligned with key transitions in feeding patterns as well as immune and microbial maturation (Hill and Spergel, 2018; Stewart et al., 2018). In this study, we observe that differences in gut microbiome between infants with allergic disease and healthy controls are minimal before 6 months of age, but become more pronounced thereafter. This indicates that the first six months of life may represent a critical window for interventions aimed at preventing the development of allergic disease.

## Materials and methods

### Study cohort

All procedures were approved by the Sterling Institutional Review Board (Atlanta, GA; IRB #10472). Participants were actively recruited between 2022 and 2024 under an IRB-approved study conducted by Tiny Health. The study enrolled children aged 0–36 months across the United States. Parents or guardians provided written informed consent and completed detailed questionnaires on medical history, feeding practices, and immune-related diagnoses.

Children with recent antibiotic or probiotic use (within two months) were excluded to minimize confounding effects on microbial maturation. For participants who self-reported immune-related or gastrointestinal reaction conditions, physician documentation was collected to confirm diagnoses. Eligible diagnoses included atopic dermatitis (AD), cow’s milk protein allergy (CMPA), IgE- and non-IgE-mediated food allergies (e.g., FPIES, EoE, FPAIP, FPE, celiac disease), lactose intolerance, and breastmilk-associated food sensitivities, defined as non-IgE food reactions causing gastrointestinal or extra-intestinal symptoms. No records were collected for participants without the above conditions.

**Figure 1:**
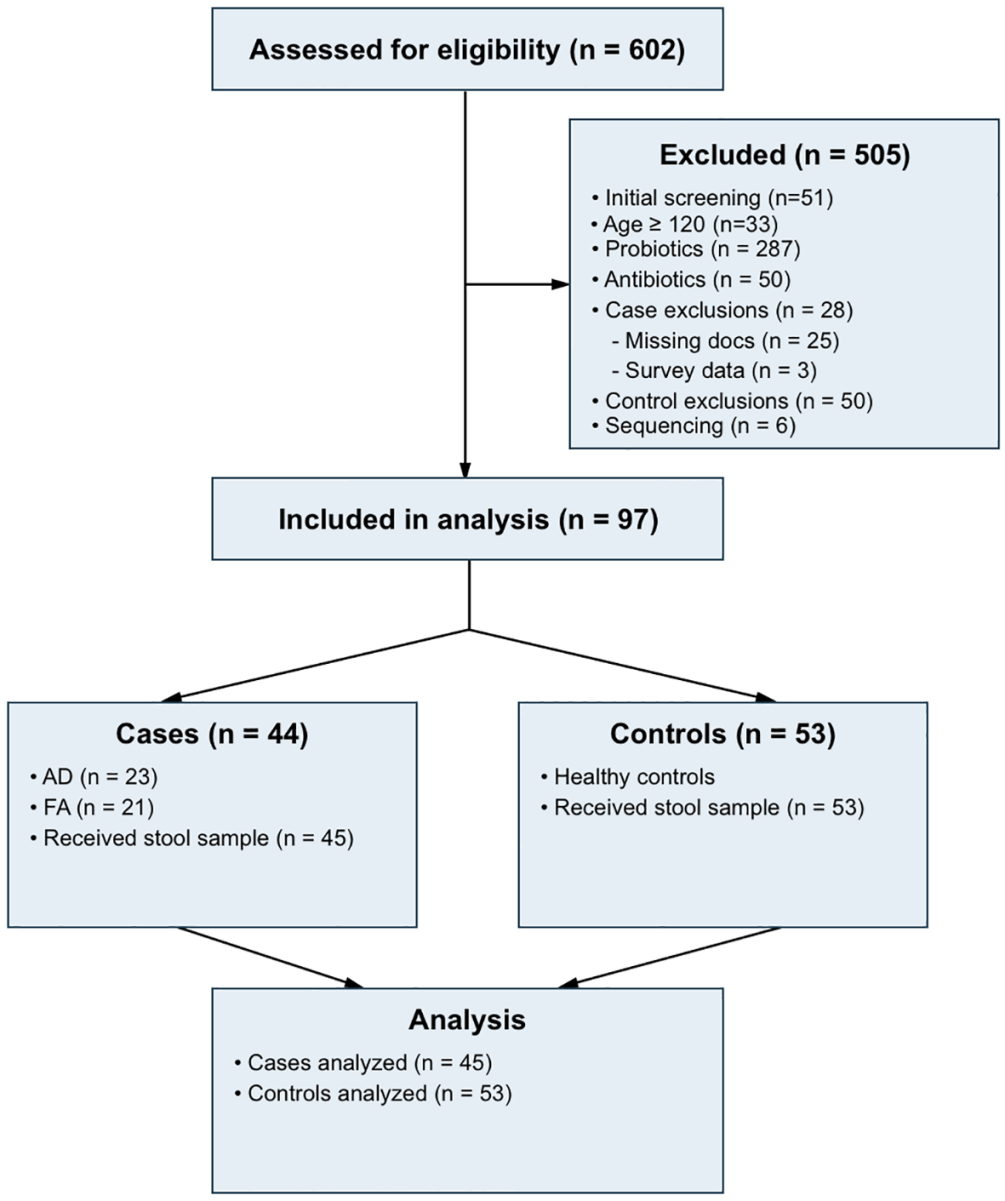
Study consort diagram. Flow diagram illustrating participant selection, sample collection, and analytical workflow in the present study, from initial eligibility assessment through final inclusion in case and control groups.

Of 602 children initially recruited, 179 reported allergic or gastrointestinal conditions and 372 reported none. After applying exclusion criteria (initial screening n=51, antibiotic n=50; probiotic n=287; unverified or incomplete documentation n=25; complete survey data n=3; control confirmation n=50, age ≥ 120 days n=33, sequencing n=6), the final analytic cohort included 97 children: 23 with eczema, 21 with food allergy, and 53 without eczema or allergies (also referred to as healthy controls). All cases were physician-confirmed and aged 4 to 36 months. We further stratified participants into three developmental stages, infancy (4–6 months), mid-infancy (6–12 months), and toddlerhood (12–36 months), to capture age-specific differences in microbiome maturation and allergy onset. **Figure 1** summarizes participant flow and exclusion criteria.

### Sample collection and shipping

Caregivers collected fecal samples using standardized stool collection kits (Copan 4N6 FLOQSwabs™ Genetics with active drying tube) provided under the study protocol. Completed kits were returned to the CAP/CLIA-certified sequencing laboratory via prepaid mailers within five days of sample collection.

### DNA extraction

DNA was extracted from the stool samples using the Qiagen Powersoil Pro kit (Qiagen, Hilden, Germany) following the manufacturer’s instructions. Briefly, microbial cells in stool samples were homogenized and lysed using mechanical disruption. The lysate was then subjected to a series of binding, washing, and elution steps to isolate high-quality microbial DNA. DNA concentration and purity were assessed using NanoDrop spectrophotometer and a Qubit™ dsDNA BR Assay Kit (Thermo Fisher Scientific Inc., Waltham, MA, United States).

### Shotgun metagenomic sequencing

Shotgun metagenomic sequencing libraries were prepared using the Nextera DNA Flex Library Prep Kit (Illumina, San Diego, CA, USA). Libraries were quantified using Qubit Fluorometer (Thermo Fisher Scientific), size distribution assessed using an Agilent TapeStation 2200 or 4200 (Agilent Technologies, Santa Clara, CA, USA), and sequencing was performed on Illumina instrument (Illumina, San Diego, CA, USA) with 150 bp paired-end chemistry. Samples were sequenced to a target of 10 million paired-end reads (actual range 6.95M-86.81M with a median read depth of 14.33M).

### Bioinformatics analysis

Raw sequencing reads were assessed for quality and trimmed low-quality reads along with adapter sequences using fastp (v0.23.4) (Chen, 2023). Duplicate reads were removed using clumpify from BBmap (v.39.06) (Bushnell, 2014). To eliminate human DNA contamination, reads were aligned to the human genome reference (GRCh38) using Bowtie2 (v2.4.2) and mapped reads were discarded (Langmead and Salzberg, 2012). Final non-human, deduplicated, trimmed read depths ranged from 5.58M-57.69M with a median read depth of 10.89M.

For taxonomic classification, cleaned reads were processed using Kraken2 (v2.1.2) with a custom database based on the GTDB database naming structure (v202) (Wood et al., 2019; Parks et al., 2022). Relative abundances of microbial taxa were calculated using Bracken (v2.6.0) (Lu et al., 2017). To reduce the potential effect of sequencing depth on gene abundance results, samples with more than 15 million reads were randomly subsampled down to 15 million reads using seqtk (v.1.4) (Li, 2016). For functional gene annotations, reads were assembled and translated into protein sequences using Plass (Release 4-687d7) (Steinegger et al., 2019). These protein sequences were then annotated with dbcan (v.3.0.2) with its associated HMM database (V10) and with the KEGG database using kofamscan (v.1.3.0) (Aramaki et al., 2020). Reads were mapped to the annotated protein sequences using MMseqs2 (v.15-6f452+ds-2). Gene counts were normalized into Reads per Kilobase Million (RPKM) using custom scripts (Mirdita et al., 2021).

### Statistical analysis

We performed all statistical analyses in R (v4.5.1) (R Core Team, 2025). We stratified analyses by three distinct age groups (4–6 months, 6–12 months, and 12–36 months). Given the modest sample size within age strata, analyses focused on identifying developmentally consistent patterns across taxonomic, functional, and composite metrics, rather than exhaustive feature discovery.

To assess overall differences in gut microbial community structure between cases and controls, we calculated Bray-Curtis dissimilarity. We used non-metric Multidimensional Scaling (NMDS) to visualize community structure. We tested for significant differences within each age stratum between conditions using Permutational Multivariate Analysis of Variance (PERMANOVA), and tested for homogeneity of multivariate dispersions using PERMDISP2. We performed both statistical tests using the vegan package in R with 9,999 permutations (Oksanen et al., 2001).

To identify specific taxa and genes associated with allergic conditions, we performed multivariable association testing separately for each age group using MaAsLin3 (v1.0) (Nickols et al., 2026). We applied several filtering steps prior to analysis. First, we set taxa relative abundances that were below 0.05% to 0 to reduce false positive detection of spurious low-abundance signals and improve reproducibility (Goldman et al., 2025; Van Uffelen et al., 2024). We then retained taxa and genes only if they were present in four or more samples, and removed features with near-zero variance using the nearZeroVar function from the caret package (Kuhn, 2008). For modeling, taxonomic abundances were TSS normalized and log-transformed, while gene abundances (already normalized in RPKM) were log-transformed.

We ran MaAsLin3 generalized models to identify microbial features associated with allergic conditions while adjusting for covariates (Nickols et al., 2026). Each model included delivery mode and non-host read count included as fixed-effect covariates. False discovery rate (FDR) correction using the Benjamini–Hochberg method was applied to account for multiple comparisons within each model. However, no associations remained significant after FDR adjustment (q < 0.1), likely reflecting the modest sample size within age strata. Therefore, nominal p-values (p < 0.05) are reported to highlight potential patterns in this exploratory analysis.

### Sensitivity analyses

To assess robustness, we repeated the age-stratified MaAsLin3 models with additional covariates (full model): sex, race/ethnicity, diet composition (solid food exposure in 4–6 and 6–12 months only), pet exposure, and sibling status. Results were compared to the primary models. Covariate effects were further evaluated using Leave-One-Out (LOO) and Add-One-In (AOI) approaches, based on changes in the number of significant features (p < 0.05).

### Genus–function correlation networks

To identify potential taxonomic contributors to SCFA biosynthesis, we constructed genus–KO co-correlation networks within each age stratum. Features present in ≥20% of samples were retained. Spearman correlations were computed between genus abundances and KO counts (RPKM), with FDR correction applied. SCFA-related KOs (butyrate, propionate, acetate) were used to define sub-networks. Significant associations (q < 0.05, |ρ| ≥ 0.50) were visualized as bipartite networks using igraph and ggraph with Fruchterman–Reingold layouts.

### Composite metrics analysis

We evaluated composite gut microbiome metrics (n = 15) to summarize groups of functionally related genes, pathways, and ecological features into interpretable indices representing microbial diversity, metabolic capacity, and inflammatory potential. These metrics included diversity measures (species richness, Shannon diversity), host-read content (Host DNA), microbial maturation (Maturation Index), SCFA-associated pathways, carbohydrate-degradation pathways (cellulose, pectin, 2′-fucosyllactose, and sialyllactose capacity), antibiotic resistance-related measures (ARO Richness and ARO Abundance indices), and inflammation- or redox-related pathways (mucus degradation index, Hexa-LPS index, and hydrogen sulfide index). Below, we describe each metric in detail.

Species richness was defined as the total number of microbial species detected per sample. Shannon diversity captured both microbial richness and evenness. Host DNA represents the percentage of sequencing reads aligning to the human reference genome relative to total non-host reads.

We calculated functional gene metrics by aggregating the relative abundance of KEGG Orthologs (KOs) and/or CAZyme families associated with specific microbial functions (Kanehisa et al., 2025; Drula et al., 2022). The pathways evaluated included acetate, propionate, butyrate production capacity, cellulose, pectin, 2′-fucosyllactose (2’-FL), and sialyllactose.

Antibiotic resistance richness and abundance indices quantified the number and relative abundance of antibiotic resistance genera, respectively. We also evaluated three inflammation-related indices. The Hexa-LPS index reflects taxa and genes related to hexacylated lipopolysaccharide (Hexa-LPS) biosynthesis. The hydrogen sulfide index represents taxa and genes involved in cysteine and sulfate reduction pathways. The mucus degradation index combines species known to degrade mucin with a curated set of glycoside hydrolase (GH) families involved in the breakdown of mucin- and glycan-derived carbohydrates.

The maturation index reflected the balance between microbial taxa characteristic of mature versus early-stage infant gut communities and incorporated genus-level taxonomic abundances, selected functional gene categories, and Shannon diversity. We adjusted this value for each individual’s age by calculating the base-2 logarithm of the raw index value divided by actual age in days.

We performed statistical analyses of all composite metrics using the non-parametric Wilcoxon rank-sum test, comparing cases to controls, as well as separate analyses for the atopic dermatitis and food allergy groups. Nominal significance was defined as p < 0.05, and p-values were adjusted for multiple testing using the Benjamini–Hochberg FDR procedure (q < 0.10).

## Results

### Participant demographics and clinical characteristics

The final analytic cohort included 97 children, including a subgroup of 44 cases with documented early-life allergic conditions (n=23 with atopic dermatitis and n=21 with food allergy) and a subgroup of 53 children without eczema or allergies (i.e. healthy controls) (**Figure 1**). Demographic and clinical characteristics were comparable across groups apart from ethnicity, which differed significantly (**Table 1**). When stratified by age, delivery mode, feeding type, and case-control distributions remained balanced within each bin (p > 0.1 for all comparisons) (**Supplementary Tables 1–3**). Stratifying participants into infancy (4–6 months), mid-infancy (6–12 months), and toddlerhood (12–36 months) allowed assessment of microbiome differences across distinct stages of development. Eczema predominated in the youngest infants, while food allergies emerged after six months of age, consistent with typical patterns of allergic disease onset (Hill and Spergel, 2018). Across age bins, approximately half of all cases presented with eczema alone (**Supplementary Figure 1**). All association analyses were adjusted for multiple comparisons, and unless otherwise stated, no associations remained significant after correction (q < 0.1); therefore, results are presented using nominal p-values for exploratory interpretation.

**Table 1:** Demographic and health characteristics of study participants.

Characteristic	Overall	Controls N = 53	AD N = 23	FA N = 21	p-value
Age (months)^†^	13.5 (8.6)	13.6 (8.9)	11.2 (8.7)	15.6 (7.7)	0.064
Sex^‡^					0.6
F	47 (48%)	25 (47%)	10 (43%)	12 (57%)	
M	50 (52%)	28 (53%)	13 (57%)	9 (43%)	
Race/Ethnicity^‡^					0.009
Other	29 (30%)	9 (17%)	10 (43%)	10 (48%)	
White	68 (70%)	44 (83%)	13 (57%)	11 (52%)	
Residential Setting^§^					0.3
Rural	12 (12%)	8 (15%)	1 (4.3%)	3 (14%)	
Suburban	63 (65%)	36 (68%)	13 (57%)	14 (67%)	
Urban	22 (23%)	9 (17%)	9 (39%)	4 (19%)	
Delivery Mode^‡^					0.6
C-section	33 (34%)	17 (32%)	7 (30%)	9 (43%)	
Vaginal	64 (66%)	36 (68%)	16 (70%)	12 (57%)	
Majority Feeding Method^§^					0.12
Breastmilk	38 (39%)	18 (34%)	14 (61%)	6 (29%)	
Formula	18 (19%)	9 (17%)	4 (17%)	5 (24%)	
Solids	41 (42%)	26 (49%)	5 (22%)	10 (48%)	
Pet Exposure^‡^					0.7
Y	36 (37%)	18 (34%)	10 (43%)	8 (38%)	
N	61 (63%)	35 (66%)	13 (57%)	13 (62%)	
Has Siblings^‡^					0.8
Y	49 (51%)	26 (49%)	13 (57%)	10 (48%)	
N	48 (49%)	27 (51%)	10 (43%)	11 (52%)	

^†^ Kruskal-Wallis rank sum test; ^‡^Pearson’s Chi-squared test; ^§^Fisher’s exact test.

### Microbial community structure is primarily shaped by age

**Figure 2:**
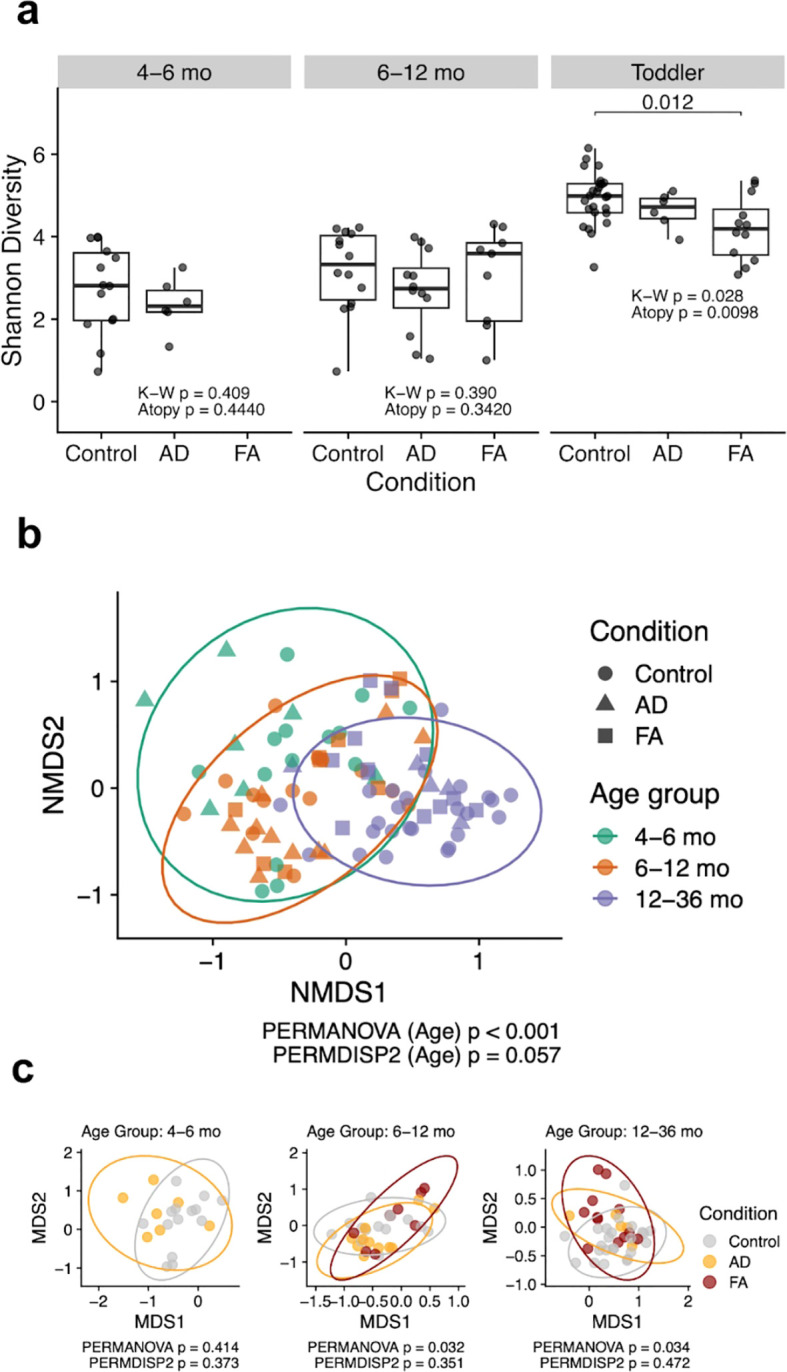
Age- and condition-dependent patterns in microbial diversity and community structure. **(A)** Shannon diversity across controls, atopic dermatitis (AD), and food allergy (FA) within each age group (4–6 months, 6–12 months, 12–36 months). Each point represents an individual sample. Within each age group, overall differences among conditions were assessed using the Kruskal-Wallis test (K-W), and controls were compared with all allergic conditions combined (AD+FA) using Wilcoxon rank-sum tests (“Cases”). When the K-W test indicated significant group-level differences, pairwise Wilcoxon tests (Control vs AD; Control vs FA) were performed, with only significant pairwise comparisons annotated. All p-values are unadjusted. **(B)** Non-metric multidimensional scaling (NMDS) ordination of Bray-Curtis dissimilarity for all samples, illustrating overall community structure across age groups and conditions. Points are colored by age group and shaped by condition; ellipses represent 95% confidence intervals for each age group. **(C)** Age-stratified NMDS plots showing community structure within each developmental stage. Points are colored by condition with 95% confidence ellipses for each condition. PERMANOVA (adonis2) p-values indicate differences in community composition, and PERMDISP2 p-values report homogeneity of multivariate dispersion, both unadjusted.

We first assessed overall gut microbial diversity between children with and without allergic conditions. Alpha diversity, measured by the Shannon diversity index, significantly increased with age, reflecting progressive microbiome maturation from infancy through toddlerhood (Spearman rank correlation ⍴=0.77, p < 0.001). During infancy, diversity metrics were comparable between children with allergic cases and without allergic conditions (i.e. controls) (all p > 0.05). However, by the toddler stage, children with allergic conditions had significantly lower Shannon diversity (cases vs. controls Wilcoxon rank sum p = 0.0057, AD vs FA vs Controls Kruskal-Wallis p = 0.022), with a decreased but non-significant trend between AD and controls (p = 0.11), and a significantly lower alpha diversity in children with food allergy compared with healthy controls in the same age range (p = 0.012) (**Figure 2A**).

Non-metric multidimensional scaling (NMDS) using Bray-Curtis dissimilarity demonstrated a clear distinction between infants and toddlers, whereas samples from cases and controls showed no distinct separation at any developmental stage (**Figure 2B**). A PERMANOVA model of the interaction between age groups and atopic conditions indicated that age explained the largest proportion of variance in microbial composition, though associations were modest (age group R^2^ = 0.11, p <0.001). The contribution of having an allergic condition to overall variation was then assessed by age group. Significant differences between control, AD, and FA groups were observed in the 6–12 month group (R^2^ = 0.1, p = 0.035) and 12–36 month group (R^2^ = 0.07, p = 0.013), while no significant effects were detected in 4–6 month infants (R^2^ = 0.05, p > 0.1) (**Figure 2C**). These findings suggest variations in microbiome structure were primarily explained by age, with only more modest differences observed between cases and controls.

### Lower relative abundance of butyrate producers and higher levels of *Enterobacteriaceae* in allergic conditions

**Figure 3:**
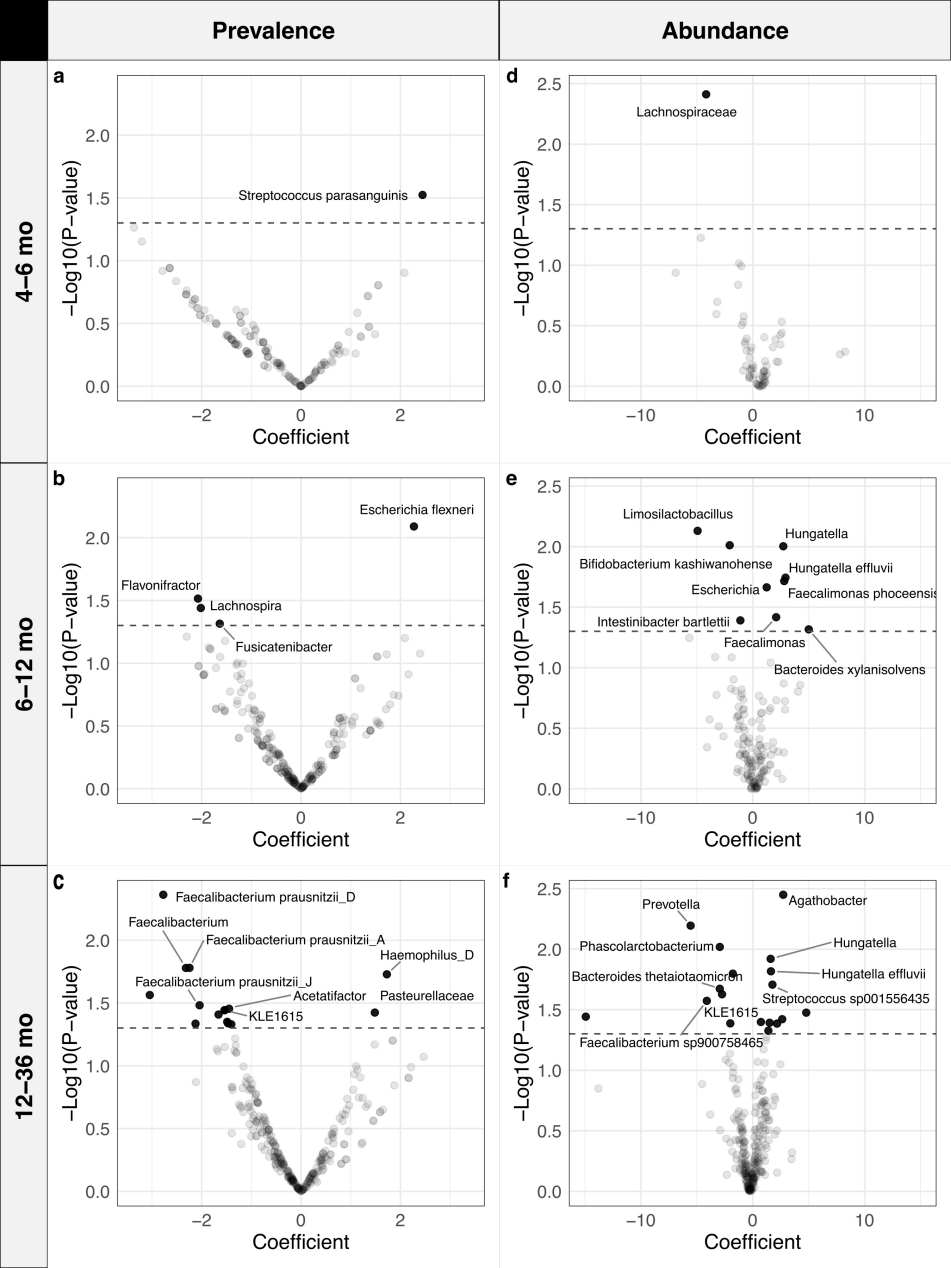
Volcano plots of taxonomic associations with allergic conditions across age groups. Volcano plots display associations between microbial features and allergic conditions across three age groups, based on the combined model. Each point represents a taxon, plotted by model coefficient (x-axis) and -log10(p-value) (y-axis). Features to the right indicate higher abundance or prevalence among allergic cases; features to the left indicate lower abundance or prevalence among allergic cases. Horizontal dashed lines denote the nominal significance threshold. **(A–C)** show prevalence-based associations at 4-6, 6-12, and 12–36 months; **(D–F)** show abundance-based associations in the same age groups. Reported associations reflect nominal significance (p < 0.05); no features remained significant after multiple testing correction (q < 0.1).

We next examined associations of both taxonomic prevalence and abundance with cases in combined (i.e., allergic conditions vs controls) and separate models (i.e., AD vs controls and FA vs controls). In the combined model, the number of associations rose with age, from 2 at 4–6 months to 13 at 6–12 months and 29 at 12–36 months (**Figure 3**, **Supplementary Table 4**). These associations are based on nominal p-values, as no taxonomic features remained significant after multiple testing correction (q < 0.1) within age-stratified models. When AD and FA were assessed on their own, more signals appeared, including features not detected in the combined analysis. In these models, we found 24 associations at 6–12 months and 32 at 12–36 months. Taken together, these findings suggest that some microbial shifts are shared across allergic conditions, while others differ between eczema and food allergy.

At 4–6 months, taxonomic differences were minimal. Since FA cases were not present in this age group, only AD was modeled against controls. Infants with AD exhibited lower abundance of the family *Lachnospiraceae* and higher prevalence of the species *Streptococcus parasanguinis* (**Figure 3**; **Supplementary Table 4**).

By 6–12 months, more taxa were significantly associated with allergic conditions. In the combined model, infants with allergic conditions showed higher relative abundance and prevalence of facultative or inflammation-associated taxa across multiple taxonomic levels, including higher prevalence of *Escherichia flexneri*, alongside lower abundance of mucosal-associated commensals such as *Limosilactobacillus*. We also observed reduced prevalence of several fiber-fermenting genera, including *Flavonifractor*, *Lachnospira*, and *Fusicatenibacter*, as well as lower prevalence of *Klebsiella* despite overall higher *Enterobacteriaceae* levels (**Figure 3**; **Supplementary Table 4**). In the FA group inflammation-associated taxa were elevated, with *E*. *flexneri* showing higher prevalence and *Clostridioides difficile* showing higher relative abundance. FA cases also showed increased prevalence of gut commensals such as *Phocaeicola vulgatus*, but reduced abundance of select *Bifidobacterium* species, including *B. breve* (**Supplementary Figure 2**; **Supplementary Table 4**). In contrast, AD exhibited milder shifts. AD cases had increased prevalence of *E. flexneri* and higher abundance of *Bifidobacterium infantis* (**Supplementary Figure 2**; **Supplementary Table 4**). *Fusicatenibacter saccharivorans* displayed opposite patterns across conditions, lower abundance in FA but higher in AD, pointing to divergent associations within this genus. Taken together, these results suggest that by mid-infancy, infants with allergic conditions tended to show higher levels of facultative or potentially inflammatory taxa and reduced representation of fiber-fermenting and mucosal-associated commensals.

By the toddler stage (12–36 months), microbial alterations became more extensive spanning multiple taxonomic ranks. In the combined model, children with allergic conditions showed higher abundance of inflammatory or mucin-utilizing taxa, including *Escherichia* and *Ruminococcus gnavus* (**Figure 3**; **Supplementary Table 4**). Consistent with mid-infancy patterns, toddlers with allergies also exhibited reduced abundance of key butyrate-producing and fiber-fermenting taxa, including *Faecalibacterium* and *Prevotella*, *Gemmiger*, *Phascolarctobacterium*, and *Bacteroides thetaiotaomicron*, indicating a potential deficit in polysaccharide fermentation capacity (**Figure 3**; **Supplementary Table 4**).

When analyzed separately, AD and FA displayed distinct but overlapping patterns. AD toddlers showed reductions in fiber-fermenting taxa such as *Phascolarctobacterium* and *Prevotella*, alongside higher abundance of commensal genera including *Agathobacter* and the emerging taxon UBA9502 (**Supplementary Figure 2**; **Supplementary Table 4**). At the same time, FA cases were enriched in inflammation-associated taxa such as *R. gnavus*. Overall, toddlers with food allergy showed the greatest number of distinct taxonomic alterations, whereas AD cases displayed more within-group variability.

### Differences in energy metabolism and amino acid pathways across allergic conditions

**Figure 4:**
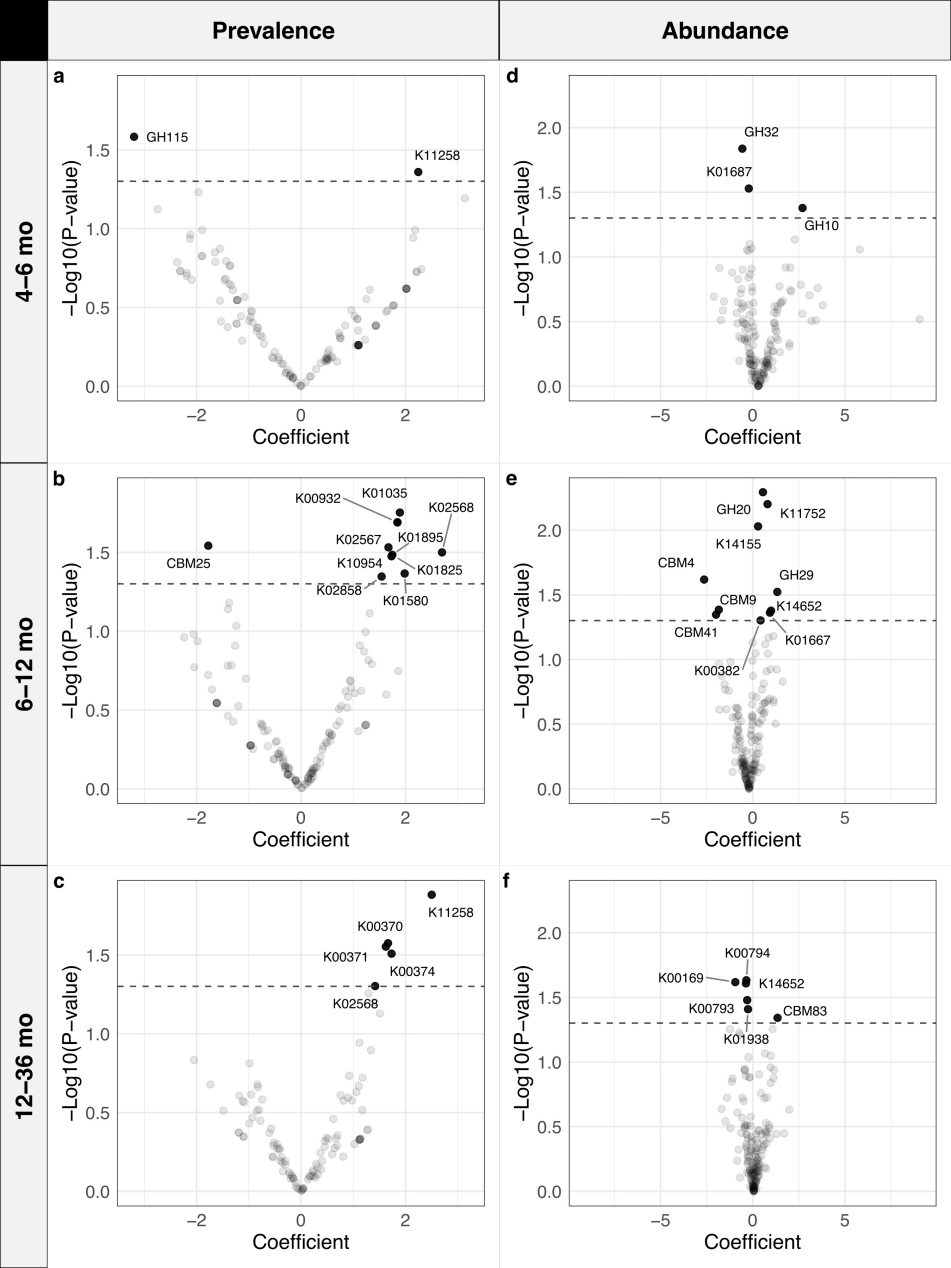
Functional gene associations with allergic conditions across age groups. Volcano plots display associations between microbial functional features and allergic conditions across three age groups, based on the combined model. Each point represents a KEGG Ortholog or CAZyme family, plotted by model coefficient on the x-axis (direction and magnitude of association) and -log10(p-value) on the y-axis (strength of evidence). Features to the right indicate functions enriched in children with allergic conditions, whereas features to the left indicate functions depleted relative to healthy controls. Horizontal dashed lines denote the nominal significance threshold. All results reflect the case-control comparison without stratifying by specific allergic condition. **(A–C)** show prevalence-based associations at 4-6, 6-12, and 12–36 months; **(D–F)** show abundance-based associations in the same age groups.

We next examined functional gene associations with early-life allergy across joint and separate models. Similar to the taxonomic findings, the magnitude and diversity of functional shifts varied by age, with fewer differences detectable at 4–6 months, and broader metabolic changes emerging by 6–12 months and in toddlers (12–36 months) (**Figure 4**; **Supplementary Table 4**). As with taxonomic results, these functional associations did not remain significant after multiple testing correction (q < 0.1) and are therefore reported at the nominal level. Modeling AD and FA separately revealed distinct patterns of higher and lower relative abundance across microbial functions, showing both shared and specific alterations in microbial metabolic potential.

In the AD group, infants at 4–6 months were characterized by reduced abundance of invertase/inulinase (GH32) and dihydroxy-acid dehydratase (K01687), an enzyme involved in branched-chain amino acid (BCAA) biosynthesis (**Figure 4**). A decrease was also observed in α-glucuronidase (GH115), which participates in the breakdown of complex plant polysaccharides. In contrast, endo-1,4-β-xylanase (GH10) was increased, indicating a potential increase in xylan degradation capacity. Together, these shifts suggest early functional alterations in carbohydrate utilization within the developing gut of infants with AD.

Consistent with the taxonomic patterns, functional differences became more pronounced by 6–12 months. In the joint model, infants with allergic conditions showed increases in genes involved in carbohydrate degradation and amino acid metabolism (e.g., GH20, GH29, K14155, K11752, K14652, K00382, K01667), suggesting greater genetic potential for nutrient breakdown and cofactor biosynthesis (**Figure 4**; **Supplementary Table 4**). Pathways contributing to SCFAs formation were more prevalent in allergic conditions, including enzymes for acetate, propionate, and butyrate production (K01035, K00932, K01895), alongside an increase in nitrate reduction capacity (K02567, K02568), indicating increased genetic capacity for fermentative and anaerobic metabolism. Conversely, carbohydrate-binding modules (CBM4, CBM9, CBM25, CBM41) were reduced, implying lower potential for microbial degradation of complex polysaccharides such as starch, cellulose, and glucans.

When modeled separately, AD infants displayed stronger representation in amino acid and cofactor metabolism (riboflavin- and tryptophan-related pathways, K11752, K14652, K01667) and glycan-modifying enzymes (GH20, GH33, GH95) (**Supplementary Figure 3**; **Supplementary Table 4**). In contrast, FA infants showed reduced abundance of glycan-binding and processing functions (CBM4, GH85, CBM25) but higher representation of genes involved in fermentative and reductive energy metabolism (K01035, K00932, K05396, K02567). Overall, these findings indicate that functional differences between cases and control infants become more apparent by 6–12 months, reflecting divergence in metabolic and carbohydrate-processing capacities prior to toddler age. These age-dependent patterns are consistent with early stages of the atopic march, in which eczema often precedes the development of food allergies.

By 12–36 months, functional differences remained. In the joint model, toddlers with allergic conditions showed increased abundance of CBM83, a module associated with starch and glycogen binding, and higher prevalence of genes involved in BCAA synthesis and nitrate reduction (K11258, K00370, K00371, K00374, K02568) (**Supplementary Figure 3**; **Supplementary Table 4**). In contrast, there was reduced abundance of several genes linked to riboflavin and folate metabolism (K00793, K00794, K14652, K01938) and central carbon metabolism and energy production (K00169, K00370). When modeled separately, the FA group showed higher relative abundance of CBM41 and K11258, consistent with elevated carbohydrate-binding and amino acid biosynthetic potential, but reduced abundance of multiple genes involved in cofactor biosynthesis, fatty acid β-oxidation, and acetyl-CoA formation (K00793, K00794, K14652, K01692, K00169, K01938, K00074, K00626, K02231, K23351, K00172, K00248). The AD group exhibited a single significant increase in GH53, a β-1,4-galactanase involved in plant-derived carbohydrate degradation. By toddler age, only a limited set of functional differences remained, mainly related to amino acid and carbohydrate metabolism.

### Adjusting for additional clinical and demographic variables

We subsequently wanted to test the influence of adjusting for additional clinical and demographic variables on these results. Sensitivity analyses incorporating additional covariates preserved most results but attenuated some associations (**Supplementary Figure 4**; **Supplementary Table 5**). Robust signals were mostly seen in older age groups. At 6–12 months, a limited set of taxa (*Escherichia*, *Hungatella*) and functions (e.g., GH20, K11752) remained significant. By 12–36 months, a broader set persisted, including *Faecalibacterium* (including *F. prausnitzii*), *Agathobacter*, and *Bacteroides thetaiotaomicron*, alongside *Clostridioides difficile* and *Streptococcus*, and functional pathways related to central metabolism and cofactor biosynthesis (e.g., K00793, K00794, K14652, K00169, K01938) and carbohydrate degradation (GH53). LOO and AOI analyses showed that diet and race/ethnicity had the largest impact on model outputs (**Supplementary Figure 4**; **Supplementary Tables 6, 7**). Inclusion of diet affected ~60% of features, and race/ethnicity ~40%, while other covariates had smaller effects (<20%).

### Genus–SCFA networks identify taxonomic sources of SCFA genes

**Figure 5:**
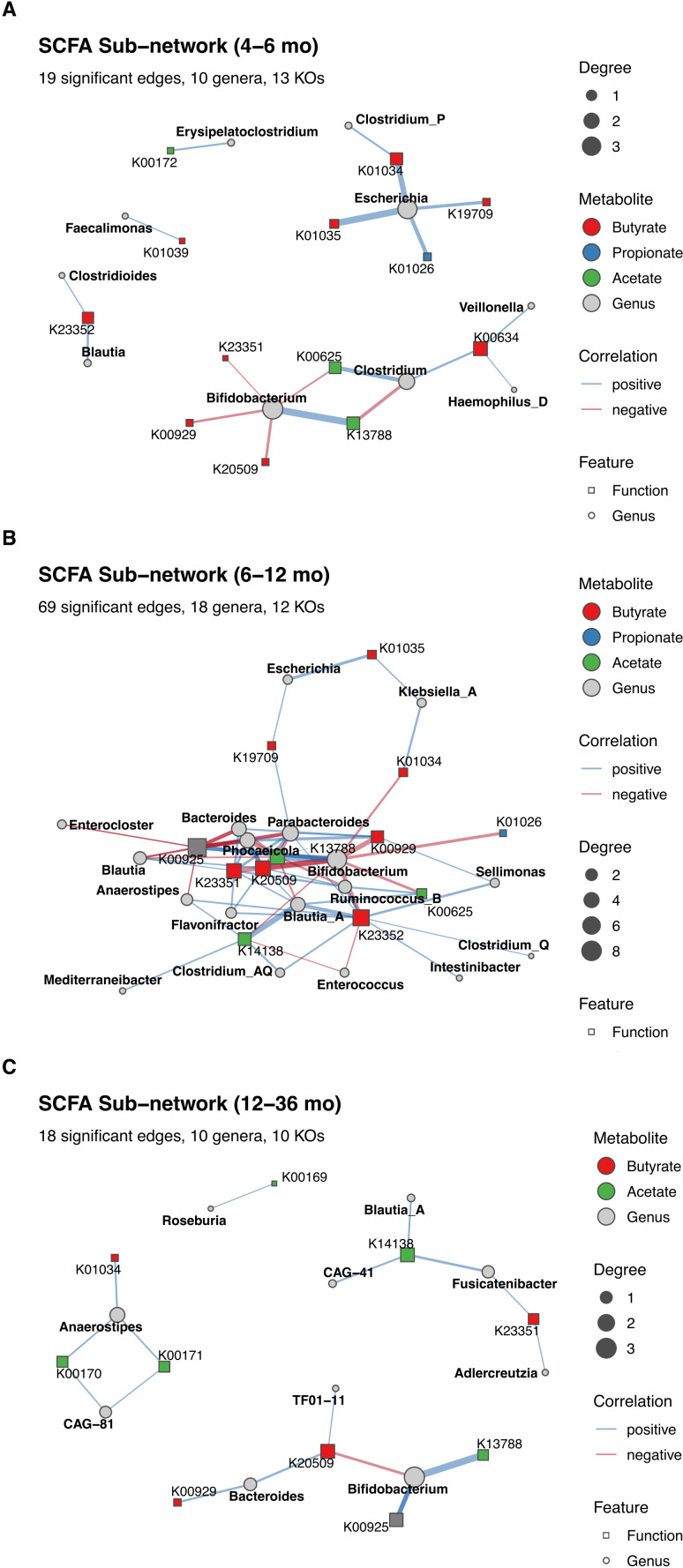
Genus–function co-correlation networks for SCFA-related pathways across early life. Bipartite networks display significant Spearman correlations between genus-level relative abundances and KEGG Orthologs (KOs) associated with short-chain fatty acid (SCFA) biosynthesis across three age groups. Nodes represent genera (circles) and KOs (squares), colored by pathway: butyrate (red), propionate (blue), and acetate (green). Edges represent significant correlations (q < 0.05, |ρ| ≥ 0.50), with color indicating direction (gray = positive, red = negative). Node size reflects degree (number of connections). **(A–C)** show networks for 4–6 months (19 edges, 10 genera, 13 KOs), 6–12 months (69 edges, 18 genera, 12 KOs), and 12–36 months (18 edges, 10 genera, 10 KOs), respectively.

The observation that SCFA biosynthesis genes were elevated in children with allergic conditions at 6–12 months, despite concurrent depletion of canonical butyrate-producing taxa, raises the question of which organisms carry these functional genes. Genus–function co-correlation networks were used to assess taxonomic attribution, computing pairwise Spearman correlations between genus-level relative abundances and KEGG Orthologs annotated to butyrate, propionate, and acetate biosynthesis (**Figure 5**, **Supplementary Table 8**). Across all age groups, *Bifidobacterium* was the strongest positive correlate of acetate pathway genes (K13788; ρ = 0.94–0.98) while showing consistent inverse correlations with butyrate pathway genes (K20509, K00929, K23351), confirming its role as an acetate producer that does not contribute to butyrate biosynthesis. At 4–6 months, *Escherichia* was the dominant positive correlate of butyrate and propionate genes (ρ = 0.79–0.92). By 6–12 months, butyrate gene associations expanded across a diverse set of commensal genera including *Blautia*, *Ruminococcus*, *Parabacteroides*, *Phocaeicola*, *Bacteroides*, and *Flavonifractor* (69 SCFA edges, 18 genera), indicating that SCFA gene carriage becomes more broadly distributed as the microbiome matures (**Figure 5**, **Supplementary Table 8**).

### Functional and composite metric differences across developmental stages

To obtain further functional insight, we evaluated age-stratified differences in composite functional metrics summarizing groups of functionally related genes or pathways, including those linked to SCFA synthesis, antibiotic resistance potential, microbial maturation, and inflammation. These metrics capture broader ecological features of the microbiome, including a maturation index that reflects how closely the gut microbiome aligns with an age-appropriate developmental profile. Analyses were conducted across the same three developmental windows and were also stratified by condition within each age group.

At 4–6 months, cases exhibited higher levels of functions associated with acetate and propionate production capacity, as well as higher antibiotic resistance gene richness and host DNA content, while displaying a lower maturation index compared to controls (p < 0.05 for all comparisons) (**Table 2**). After correction for multiple testing, propionate capacity, antibiotic resistance gene richness, and host DNA remained significant (q < 0.1) (**Table 2**).

**Table 2:** Composite and functional metrics significantly different between cases and controls.

Age	Cases (N)	Controls (N)	Metric	Cases (median)	Controls (median)	p-value	q-value
4–6 mo	7	14	Host DNA	0.79%	0.06%	0.006	0.093^*^
			Propionate capacity	1222.0	1018.1	0.010	0.093^*^
			ARO Richness Index	0.808	0.549	0.010	0.093^*^
			Acetate capacity	1566.5	1337.5	0.025	0.162
			Adjusted Maturation Index	-0.221	0.326	0.025	0.162
6–12 mo	20	14	2’-Fucosyllactose capacity	2560.0	1567.9	0.030	0.164
			Mucus degradation index	9.872	8.892	0.047	0.207
12–36 mo	17	25	Adjusted Maturation Index	-0.342	-0.158	0.002	0.093^*^
			Shannon diversity	4.334	4.989	0.006	0.093^*^
			ARO Richness Index	0.245	0.140	0.008	0.093^*^
			ARO Abundance Index	0.031	0.014	0.009	0.093^*^
			Hydrogen sulfide index	10.002	8.786	0.017	0.140
			Cellulose capacity	1974.7	1591.4	0.030	0.164
			Hexa-LPS index	9.600	9.426	0.043	0.207
			Mucus degradation index	9.497	8.349	0.048	0.207

^*^ Indicates metrics meeting both nominal significance (p < 0.05) and FDR significance (q < 0.1). Metrics without an asterisk reached nominal significance (p < 0.05) but did not meet the FDR threshold.

By mid-infancy (6–12 months), when microbial composition begins to differentiate more clearly by condition, functional disparities were also observed. In the combined case-control model, cases showed higher 2′-fucosyllactose (2’-FL) utilization capacity and greater mucus degradation potential (p < 0.05) (**Table 2**). Similarly, in the AD group, we detected higher 2’-FL and sialyllactose utilization capacity and greater mucus degradation potential relative to controls (**Supplementary Table 7**). These results suggest a potential early shift in carbohydrate metabolism functions in allergic conditions.

By toddler age (12–36 months), allergic-control differences included indices reflecting inflammatory potential and redox-active metabolism. Cases showed higher values for the antibiotic resistance richness and abundance indices, hydrogen sulfide index, cellulose and mucus degradation capacity, and Hexa-LPS index, alongside lower maturation and diversity metrics (p < 0.05). After false-discovery-rate adjustment, maturation index, Shannon diversity, and both antibiotic resistance indices remained significant (q < 0.1) (**Table 2**).

In the AD group, pectin degradation capacity was elevated compared to controls, while in the FA group, the Hexa-LPS, antibiotic resistance, and hydrogen sulfide indices were higher (p < 0.05) (**Supplementary Table 9**). These findings point to a progressive functional shift in older children with allergic conditions toward microbial communities enriched in antibiotic resistance and sulfur-metabolizing pathways, features previously linked to inflammatory stress and oxygen tolerance in the gut (Byndloss et al., 2017).

## Discussion

In this study, we examined how gut microbiome features differ between children with eczema or food allergy and non-allergic controls across early development, spanning infancy, mid-infancy, and toddlerhood. Using shotgun metagenomics, we identified when microbial and functional differences are most distinct. Differences were minimal in early infancy, became clearer by mid-infancy (6–12 months), and persisted through toddlerhood, a developmental window marked by dietary transitions, rapid microbial diversification, and immune maturation (Nunez et al., 2025).

Our findings support a shared microbial signature between eczema and food allergies (De Filippis et al., 2021; Ta et al., 2020). By mid-infancy, both conditions showed consistent depletion of butyrate-producing taxa, including *F*. *prausnitzii*, *Phascolarctobacterium*, and *Prevotella*, and enrichment of facultative or inflammation-associated taxa such as *R*. *gnavus*, *E*. coli, and *C*. *difficile*. These age-dependent patterns align with the atopic march, in which eczema often precedes the development of food allergies, and suggest that microbiome divergence during mid-infancy may coincide with this progression of allergic disease. Similar patterns have been reported in other human cohorts (Ta et al., 2020; Song et al., 2016; Candela et al., 2012). In particular, reduced *Faecalibacterium*, a major butyrate producer that promotes IL-10-mediated immune tolerance, suggests how loss of these microbes may contribute to dysregulated immune development (Song et al., 2016; Candela et al., 2012; Wopereis et al., 2018).

Functional and composite metric analyses further reflected this developmental trajectory, with functional annotations reflecting inferred metabolic potential rather than direct metabolic activity. During early infancy, cases showed elevated acetate and propionate capacity, greater antibiotic-resistance organism richness, and lower maturation index scores, an early pattern of functional immaturity and ecological imbalance. Cohort data from the ALADDIN and GUSTO studies similarly show early expansion of facultative anaerobes and high antibiotic resistance genes (ARGs) burden in infants who later develop allergic disease (Chatzigiannidou et al., 2025; Loo et al., 2020), supporting a shared facultative-dominant, ARG-rich early-life state.

By mid-infancy, disparities became more defined, with higher 2′-fucosyllactose and sialyllactose utilization capacity and greater mucus degradation potential in cases, indicating a premature shift in carbohydrate metabolism away from a typical *Bifidobacterium*-dominated, HMO-driven ecology (Henrick et al., 2021; Nunez et al., 2025). By toddler age, differences included higher hydrogen sulfide, Hexa-LPS, and antibiotic resistance indices, reflecting an increased representation of redox and inflammation-associated pathways consistent with expansion of *Enterobacteriaceae* and other oxygen-tolerant taxa in dysbiotic states (Byndloss et al., 2017; Yin et al., 2025). Reduced representation of SCFA-producing taxa, such as *Faecalibacterium* and *Phascolarctobacterium*, in line with prior reports linking loss of these producers and lower stool SCFA concentrations to increased allergy risk (De Filippis et al., 2021; Ta et al., 2020; Roduit et al., 2019). In our cohort, alpha diversity remained comparable during infancy and declined modestly only in toddlers with food allergy, consistent with evidence that overall diversity is a poor standalone predictor of allergic outcomes (Wang et al., 2022; Cheung et al., 2023). Instead, disease-specific microbial and functional signatures may be more informative. For instance, *Ruminococcus gnavus* enrichment in toddlers with food allergy in our cohort parallels findings from longitudinal studies reporting its expansion and proinflammatory pangenome in allergic disease (De Filippis et al., 2021).

*Fusicatenibacter saccharivorans* showed different patterns by condition. It was higher in eczema but lower in food allergy, which suggests that its role may vary across these two forms of allergic disease. Although this species can make acetate, it also takes part in other forms of carbohydrate use, so these different patterns likely reflect shifts in the broader gut environment rather than a single metabolic effect (Takeshita et al., 2016). Several *Bifidobacterium* species, including *B*. *breve* and *B*. *kashiwanohense*, were reduced in food allergy, consistent with prior associations between early *Bifidobacterium* depletion and allergy risk (Korpela et al., 2024; De Filippis et al., 2021).

Higher relative abundance of *Escherichia* and *C*. *difficile* in our cohort echoes earlier findings in allergic infants (Wopereis et al., 2018; Azad et al., 2015). *Klebsiella* was not elevated, despite its enrichment in some eczema cohorts (Fan et al., 2022), suggesting intra-family competition within *Enterobacteriaceae* where *Escherichia* may outcompete other facultative anaerobes (Byndloss et al., 2017).

Despite several strengths including a probiotic-naive cohort with physician-diagnosed immune conditions, age-stratified analysis across a broad early-life window, and the use of high-resolution shotgun metagenomics with standardized processing, this study has limitations. The cross-sectional design limits causal inference and prevents assessment of temporal dynamics in microbiome development. In addition, modest sample sizes within each age stratum reduce statistical power to detect small-to-moderate effects across a large number of taxonomic and functional features and may limit generalizability. Although the cohort was well-characterized, residual confounding remains possible. Sensitivity analyses incorporating additional covariates, including race/ethnicity, diet (solid food exposure), pet exposure, sibling status, and sex, indicated that some of these variables, particularly diet and race/ethnicity, influenced model outputs. This effect was most pronounced in the 6–12 month window, a period marked by substantial dietary diversification and microbiome restructuring (Mallott et al., 2023).

From a functional perspective, pathway-level inference was based on gene annotation without direct metabolite measurements, limiting conclusions about actual metabolic output. Additionally, taxonomic attribution of functional signals was inferred indirectly (e.g., co-correlation networks) rather than through assembly-based binning approaches. Importantly, no associations remained significant after FDR correction, and results based on nominal p-values should be interpreted as exploratory and hypothesis-generating. Despite these considerations, the observed patterns are broadly consistent with prior human studies of early-life atopic conditions, supporting a model in which delayed microbial maturation, characterized by reduced abundance of obligate anaerobic, butyrate-producing taxa and increased representation of facultative anaerobes, emerges during mid-infancy and persists into toddlerhood. These findings, together with evidence of distributed functional capacity across microbial communities, reinforce the importance of developmental timing in microbiome–immune interactions.

In conclusion, our age-stratified metagenomic analysis identifies mid-infancy (6–12 months) as the period when gut microbial divergence between allergic and non-allergic children becomes most apparent, with differences extending into the toddler years. These findings align with and extend prior work by integrating species-level and functional insights, supporting a model in which delayed microbial maturation and expansion of facultative, inflammation-linked taxa contribute to early immune dysregulation. Future longitudinal and interventional studies will be essential to determine whether microbiome modulation strategies can be employed in this early window (<6 months) to prevent progression along the atopic march.

## Data Availability

The data that support the findings of this study are openly available in the NCBI BioProject repository under accession number PRJNA1222487.
