# Supplementary material for: Age-specific early-life gut microbiome associations with eczema and food allergies during early immune development
Source: Front Microbiomes. 2026 May 29;5:1804117. doi: 10.3389/frmbi.2026.1804117 (PMC13260514; doi:10.3389/frmbi.2026.1804117)
Supplement: Supplementary file 1 [file DataSheet1.pdf]

SUPPLEMENTARY FIGURES

Figure S1. Distribution of self-reported allergic and gastrointestinal conditions across age groups.

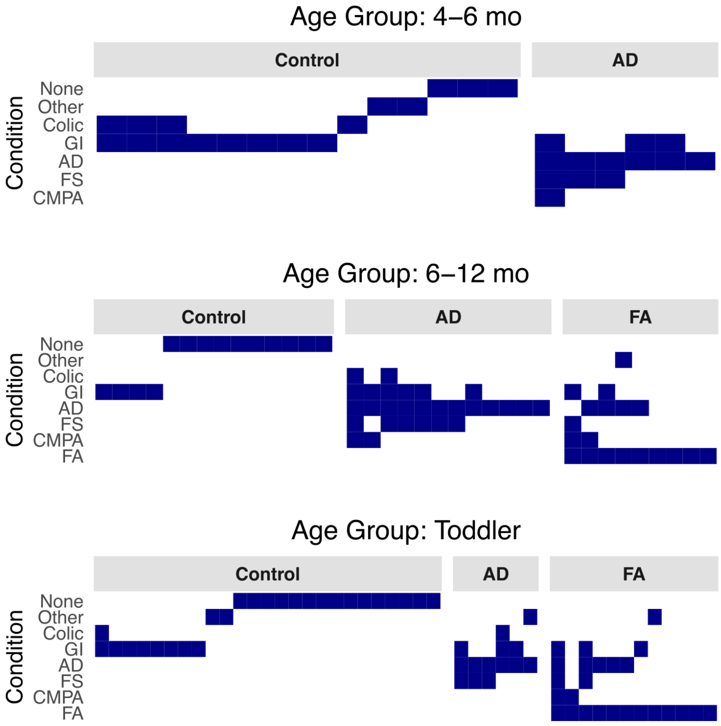

Participants were stratified into three developmental windows, 4–6 months, 6–12 months, and Toddler (12-36 months,)to account for age-related changes in gut microbiome maturation and the timing of allergic disease onset. Each panel displays a binary (presence–absence) heatmap, where dark blue blocks indicate the presence of a reported condition for a given participant within each age and diagnostic group (Control, AD, FA). Conditions shown on the y-axis include none (no reported conditions), colic, gastrointestinal symptoms (GI), atopic dermatitis (AD), food sensitivity (FS), cow's milk protein allergy (CMPA), IgE-mediated food allergy (FA), and other conditions. Reported cases of AD and FA were confirmed with physician diagnoses.

Figure S2. Volcano plots of taxonomic associations with atopic dermatitis and food allergy across age groups.

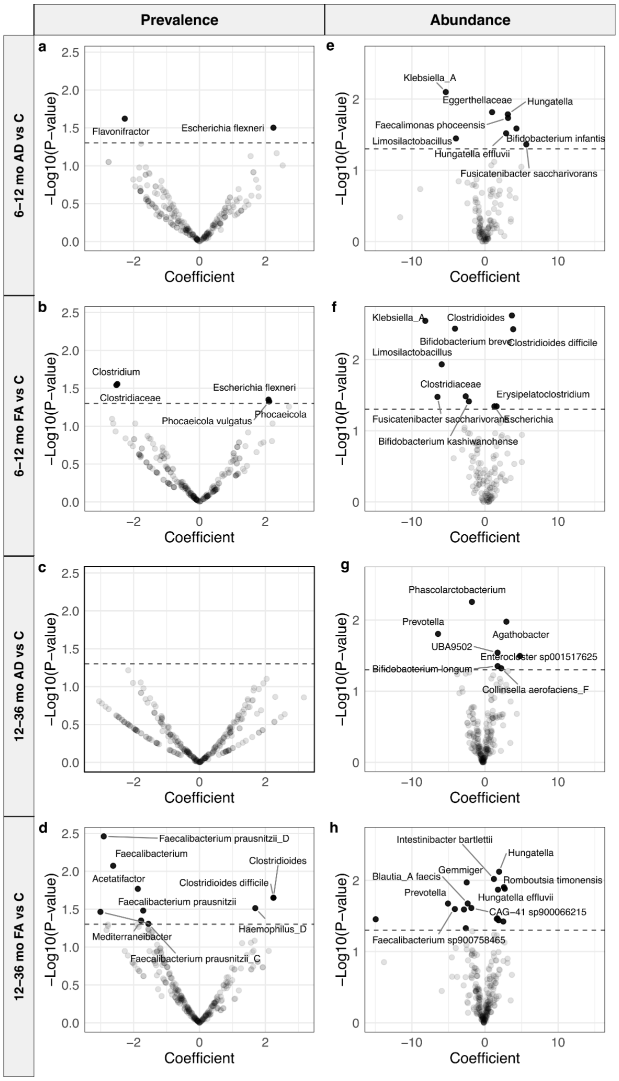

Volcano plots display associations between microbial features and allergic conditions, modeled separately for atopic dermatitis (AD) and food allergy (FA) within each age group. Each point represents a taxon, plotted by model coefficient (x-axis) and -log10(p-value) (y-axis). Features to the right indicate higher abundance or prevalence among allergic cases; features to the left indicate lower abundance or prevalence among allergic cases. Horizontal dashed lines denote the nominal significance threshold. Panels A-D show prevalence-based for AD (a, c) and FA (b, d) at 6–12 months (a, b) and 12–36 months (c, d). Panels E-H show abundance-based associations in the same allergic and age groups.

Figure S3. Volcano plots of functional associations with atopic dermatitis and food allergy across age groups.

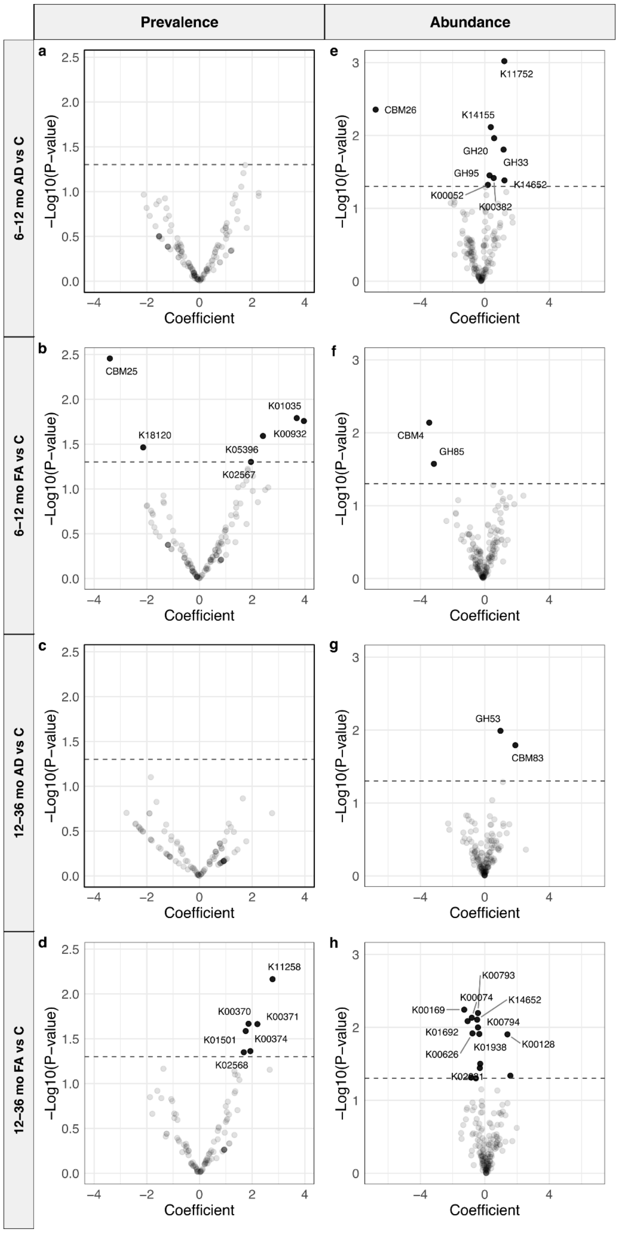

Volcano plots display associations between microbial functional features and allergic conditions, modeled separately for atopic dermatitis (AD) and food allergy (FA) within each age group. Each point represents a KEGG Ortholog or CAZyme family, plotted by model coefficient on the x-axis (direction and magnitude of association) and -log10(p-value) on the y-axis (strength of evidence). Features to the right indicate functions enriched in children with allergic conditions, whereas features to the left indicate functions depleted relative to healthy controls. Horizontal dashed lines denote the nominal significance threshold. All results reflect the case-control comparison without stratifying by specific allergic condition. Panels A-D show prevalence-based for AD (a, c) and FA (b, d) at 6–12 months (a, b) and 12–36 months (c, d). Panels E-H show abundance-based associations in the same allergic and age groups.

Figure S4. Sensitivity analyses of taxonomic and functional associations across age groups.

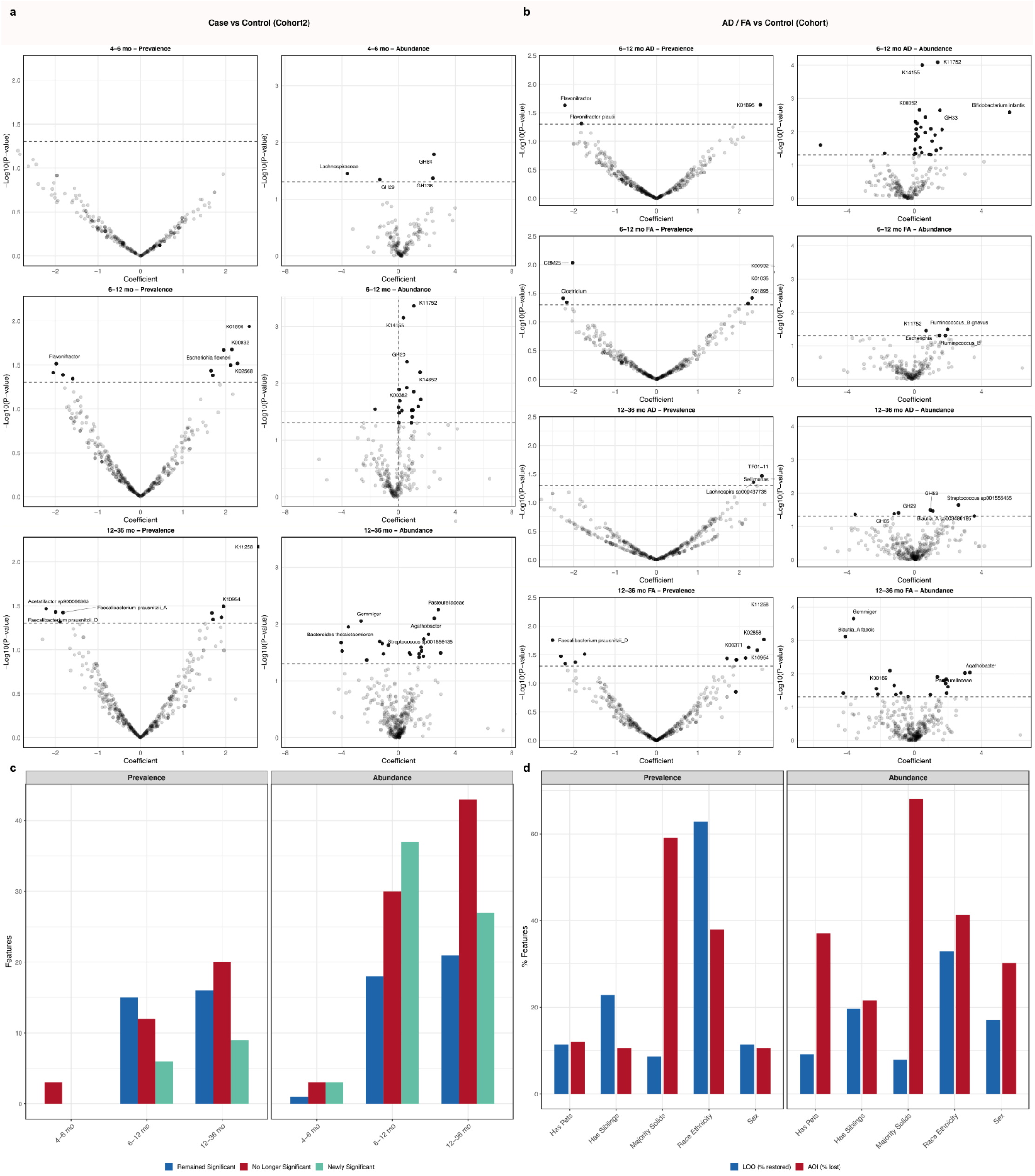

(a) Volcano plots showing associations between microbial features and allergic conditions in the full covariate model (sex, race/ethnicity, diet, pet exposure, sibling status). Points represent taxa, KEGG Orthologs, or CAZyme families, plotted by effect size (x-axis) and $-\log_{10}$(p-value) (y-axis). Prevalence (left) and abundance (right) results are shown across age groups. Dashed lines indicate $p < 0.05$. (b) Volcano plots for atopic dermatitis (AD) and food allergy (FA) vs. controls (6–12 and 12–36 months). (c) Summary of feature-level changes between primary and full models, showing features retained, lost, or newly identified after covariate adjustment. (d) Covariate impact analysis using leave-one-out (LOO) and add-one-in (AOI) approaches, showing the proportion of features restored or lost across models.
